# Encapsulation of Anthocyanins from Cornelian Cherry Fruits Using Heated or Non-Heated Soy Proteins

**DOI:** 10.3390/foods10061342

**Published:** 2021-06-10

**Authors:** Loredana Dumitrașcu, Nicoleta Stănciuc, Iuliana Aprodu

**Affiliations:** Faculty of Food Science and Engineering, Dunarea de Jos University of Galati, 800201 Galati, Romania; loredana.dumitrascu@ugal.ro (L.D.); Nicoleta.Stanciuc@ugal.ro (N.S.)

**Keywords:** cornelian cherry (*Cornus mas* L.), microencapsulation, soy proteins, anthocyanins, heating

## Abstract

In the current study, the effect of temperature on the potential of soy proteins to ensure the encapsulation and gastric stability of bioactives, such as anthocyanins from cornelian cherry fruits, was investigated. The powders obtained after freeze-drying were analyzed in relation to flow properties, encapsulation retention and efficiency, stability in simulated gastrointestinal medium, color, and morphology. Preheating the soy proteins generated a powder with low density. Powders obtained with native soy proteins allowed the highest encapsulation efficiency and the lowest was obtained when using preheated soy proteins. The heat treatment of the mixture of soy proteins and cornelian cherry fruits prior to encapsulation generated powders with the highest lightness and the lowest intensity of red shades among all samples. The in vitro experiments revealed that the highest protection in simulated gastric environment was provided when protein was heat treated either alone or in combination with bioactives to be encapsulated. The morphological analysis highlighted that powders consisted of large and rigid structures.

## 1. Introduction

*Cornus mas* L. belongs to the Cornaceae family, grown in several countries from Europe and Asia. The plant tolerates harsh conditions, being able to resist both biotic and abiotic factors for up to 300 years [1]. Many parts of this plant, such as the fruits, flowers, leaves or bark, have found applications in traditional medicine since ancient times. Depending on the cultivar, the ripening period begins at the end of summer and ends in the middle of autumn. At full maturity, the fruit color ranges from yellow, red, or black, the red color being the most predominant among the cornelian cherry varieties.

The fruits are edible, juicy, and usually have elliptic or spherical dimensions and a distinct flavor. Their taste is sour, tart sweet, and for some cultivars, is sweet-pineapple [2]. Cornelian cherry fruits are abundant in bioactive compounds such as anthocyanins, phenolic acids, flavonoids, iridoids, tannins, carbohydrates, fatty acids, vitamins, and minerals [3,4]. In the food industry, applications based on these fruits range from juices, beverages such as wine, beers, or liquors, to candies, marmalades, sauces, and syrups [5,6,7,8]. Many studies have focused on testing the biological activities of *Cornus mas* L. [1]. Polyphenolic compounds and ascorbic acid have shown high antioxidant activity. The extracts of cornelian cherry fruits and leaves have presented antimicrobial activity against several pathogens, such as *Staphylococcus aureus* [9], *Pseudomonas aeruginosa* [10], *Clostridium perfringens*, *Listeria monocytogenes*, *Salmonella enteritidis*, *Shigella sonnei* etc [11]. Recent studies showed the renal, neuro and cardio protective, hypolipidemic, anti-diabetic, anti-obesity, anti-inflammatory, anti-hypertensive, and anti-glaucoma effects of cornelian cherry fruits [1,3,4].

The most important health benefits of this plant have been associated to the glycosides of anthocyanidins, known as anthocyanins, out of which cyanidin-3-glucoside, cyanidin-3-robinobioside, cyanidin-3-O-rutinoside, peonidin-3-O-glucoside, pelargonidin-3-O-glucoside, pelargonidin-3-robinobioside, pelargonidin-3-O-galactoside, cyanidin-3-O-galactoside, delphinidin-3-O-galactoside and petunidin-3-glucoside are the most prevalent of cornelian cherry fruits [4,12,13]. On the other hand, during processing and storage, anthocyanins are negatively affected when exposed to physical (temperature, light, and oxygen) and chemical (pH and solvent) factors [14]. The stability of these compounds can be achieved through encapsulation. When encapsulated, the bioactive compounds are protected by wall materials to form microcapsules, which secure their delivery to the target system. Thus, the core and carrier material are considered the structural elements of encapsulation [15]. The type of wall material is essential, as it influences the encapsulation efficiency and powder characteristics.

Many materials have been reported for the encapsulation of anthocyanins, including proteins, maltodextrins, gums, pectins, etc. Plant proteins, such as soy, are cheap, biocompatible, biodegradable, nontoxic, highly nutritious and their functional properties such as emulsification, solubility, film-forming, and water binding capacity are ideal for encapsulation. Wu et al. [16] demonstrated that soy proteins are a functional and promising candidate for delaying anthocyanins release and preventing disease through the promotion of gut health.

The most applied methods for encapsulation are freeze-drying and spray-drying. Compared to spray-drying, freeze-drying is an excellent procedure for drying heat-sensitive compounds, as it reduces the thermal degradation reactions, being used intensively for polyphenols encapsulation [17]. Through freeze-drying, the changes in the protein structure, volatilization of different compounds, formation of resistant layers as well as the transfer of the solubilized compounds towards the surface of the product during drying are minimized [14]. Thus, freeze-drying generates high-quality products, allowing to preserve the functionality, and prolonging the shelf-life of bioactive compounds [18].

Although many studies have been conducted to identify suitable methods for stabilizing the anthocyanins, the information regarding the protective effect of soy proteins on cornelian cherry anthocyanins are limited. In a previous study, we tested the ability of soy proteins in combination with maltodextrin to encapsulate anthocyanins from cornelian cherry juice and showed that the encapsulation was dependent on the wall material as well as the drying technique [19]. The objective of the present study was to investigate the effect of the thermal treatment specific to pasteurizations and sterilization regimes on the potential of soy proteins to ensure the efficient encapsulation and gastric stability of bioactives from cornelian cherry fruits. Freeze-drying was used as the encapsulation technique and powders were analyzed in relation to microstructure, flow properties, encapsulation retention and efficiency, color properties and anthocyanins stability when subjecting the microcapsules to the simulated gastrointestinal (GI) digestion.

## 2. Materials and Methods

### 2.1. Materials

Fresh cornelian cherry fruits at full maturity (Bordo cultivar) were procured from the local market (Roman, Neamt, Romania) in August 2019. Soy protein concentrate (SPC) was distributed by Ubimedia S.R.L. (Galati, Romania) (82.1% crude protein). All other chemical reagents were of analytical grade. The pepsin and pancreatin of porcine origin, red congo fluorochrome were delivered by Sigma Aldrich (Chemie GmbH, Taufkirchen, Germany).

### 2.2. Obtaining the Cornelian Cherry Powder (CCP)

The fruits were first rinsed with tap water and freeze-dried (Christ Alpha 1–4 LD plus, Martin Christ Gefriertrocknungsanlagen GmbH, Osterode am Harz, Germany) over 72 h, at −42 °C and 0.10 mBar. The freeze-dried fruits were grounded and deposited at 4 °C until analysis (no more than one month).

### 2.3. Obtaining the Microcapsule Powders

First, 2.5 g SPC was dispersed into 100 g water and mixed thoroughly using a magnetic stirrer until the protein was hydrated. For variant S1, the SPC solution was mixed with CCP. For variant S2, SPC solution was first heated at 121 °C for 15 min using an autoclave at ~1 bar, cooled to room temperature with ice water and then mixed with CCP. Variant S3 was obtained by mixing the SPC solution with CCP, the resulting mixture being heated at 80 °C for 5 min. These temperatures selected for applying the thermal treatment on the soy proteins are specific to sterilization (121 °C) and pasteurization (80 °C) usually applied for preservation in the food industry. For all the tested variants, the final ratio between SPC and CCP was 2:1 (*w*/*w*). The samples were homogenized at 8000 rpm for 2 min (Ultra Turrax—KA T18 basic, IKA-Werke GmbH & Co., Staufen, Germany) and then freeze-dried, using the same parameters as in the case of CCP preparations.

### 2.4. Modeling the Interactions between Main Soy Proteins and Anthocyanin Molecules

The models of main soybean proteins, namely α’ homotrimer of β-conglycinin (PDB ID 1UIK, [20]), β homotrimer of β-conglycinin (PDB ID 1IPJ, [21]) and the homotrimer of glycinin consisting of three A1aB1b subunits (PDB ID 1FXZ, [22]) from RCSB Protein Data Bank were used to check the atomic level interactions with cyanidin 3-glucoside and cyanidin 3-rutinoside. For convenience, these models were further termed 7S-α, 7S-β and 11S, respectively. Following the sequence of molecular mechanics and dynamics steps indicated by Dumitraşcu et al. [23] investigating the thermal dependent behavior of 7S-α and 11S, the molecular model of 7S-β was optimized and relaxed at 25 °C using the Gromacs 5.1.1 software [24]. The molecular docking PatchDock algorithm [25] was then used to obtain the best ligand-protein fits based on shape complementarity.

### 2.5. Characterization of the Powders

The flow properties of the powders were measured as described earlier [19]. Briefly, the bulk density (BD, kg/m^3^) was estimated as the ratio between the mass of powder (2 g) placed in a 10 mL cylinder and its volume, whereas the tapped density (TD, kg/m^3^) was obtained as the ratio between the weight and volume of the powder resulting from slowly dropping the cylinder with the powder from a 15 cm height 100 times. Furthermore, BD and TP were used to calculate the Carr index (CI) and Hausner ratio (HR) [26]. CI values below 15 are specific for powders with good and excellent flowability; in the range of 15 to 25 are specific for those with fair and passable flowability, whereas CI higher than 25 are specific to powders with poor flowability. The HR index can be used for classifying the samples as free (<1.11), medium (1.11 to 1.25) and poor (>1.25) flowing powders [26].

The water content and water activity (a_w_) of the powders were determined as previously reported [19].

The encapsulation efficiency (EE) was estimated based on the total anthocyanins content (TAC) and surface anthocyanins content (SAC), as previously reported [19]. For TAC, an amount of 0.2 g of powder was mixed with the methanol:acetic acid:water (50:8:42 *v*/*v*/*v*) solvent. The extraction was assisted by ultrasonication (DCG-80H, MRC Scientific Instruments Ltd., Holon, Israel) at 40 kHz for 20 min, and the mixture was then centrifuged for 5 min 7697× *g* at 4 °C. In case of SAC, 0.2 g of powder was combined with a mixture of ethanol:methanol solution (1:1 *v*/*v*), vortexed for 1 min and centrifuged as described previously. TAC was assessed on the supernatant [4] and was expressed as mg C3G equivalents/100 g dry weight (d.w.). The retention efficiency (RE) was calculated using Equation (1), whereas EE was calculated according to Equation (2):

(1) $$\mathrm{RE}\;\left(\%\right)=\frac{\mathrm{TAC}\;\mathrm{in}\;\mathrm{the}\;\mathrm{powder}\;}{\mathrm{TAC}\;\mathrm{in}\;\mathrm{the}\;\mathrm{fruits}}\times 100$$

(2) $$\mathrm{EE}\;\left(\%\right)=\frac{\mathrm{TAC}-\mathrm{SAC}}{\mathrm{TAC}}\times 100\;$$

The Chroma Meter CR-410 (Konica Minolta Sensing Americas Inc., Ramsey, NJ, USA) was used to measure the L* (100—white; 0—black), a* (+red; −green) and b* (+ yellow; −blue) color parameters of the powders. Prior to color measurements, the white calibration was performed against the dedicated white calibration plate, in agreement with the suggestion of the producer. The powder was transferred on a glass plate and well-tapped with the clear glass window of the cell to fix the loose powder. The L*, a* and b* values were used to calculate chroma and hue angles using Equations (3)–(6) [27]:

Chroma = (a*^2^ + b*^2^)^1/2^ (3)

Hue angle = tan^−1^(b*/a*) for quadrant I (+a*, +b*) (4)

Hue angle = 180 + tan ^−1^(b*/a*) for quadrant II (−a*, +b*) and quadrant III (−a*, −b*) (5)

Hue angle = 360 + tan ^−1^(b*/a*) for quadrant IV (+a*, −b*) (6)

The color of the samples was measured immediately after freeze-drying and stability was checked after 7, 14 and 21 days of storage.

### 2.6. Confocal Microscopy Analysis

The microscopy analysis of the powders was performed using the AxioObserver Z1 inverted LSM 710 confocal microscope (Carl Zeiss, Oberkochen, Germany) and the dedicated black edition of the ZEN 2012 SP1 software. The microstructural characteristics of the powder samples were labeled with 40 μM Red Congo fluorochrome and the images were collected as in [19].

### 2.7. In Vitro Digestibility

The simulated gastric and intestinal digestion was performed as recently reported [19]. In brief, the powders were first mixed with 10 mMTris-HCl buffer of pH 7.5 (ratio of 10:1, *w*/*v*) and added further to the simulated gastric juice (SGJ) (ratio of 1:2, *w*/*v*). The SGJ consisted of porcine pepsin in 0.1 M HCl, pH 2.0 (40 mg/mL). Afterwards, the SGJ was mixed with simulated intestinal juice (SIJ, 2 mg pancreatin/mL 0.9 M sodium bicarbonate) at a ratio of 1:1 (*v*/*v*) at pH 7.0. The gastric and intestinal digestions, lasting 120 min each, were carried out by incubating the samples in an SI e300R orbital shaker (Medline Scientific, Chalgrove, Oxon, UK) at 100 rpm and 37 °C. At the end of each digestion, a step a volume of 0.2 mL mixture was collected for TAC determination, to finally estimate the percentage of anthocyanins release out of powders, in the presence of SGJ and SIJ.

### 2.8. Statistical Analysis

The results are presented as mean ± standard deviation. The differences between samples were determined based on the ANOVA test available on Minitab 19 software (Minitab Inc., State College, PA, USA) and the post-hoc Tukey test method was further performed at *p* < 0.05. All the samples passed the normality and homoscedasticity conditions.

## 3. Results and Discussion

### 3.1. Physical Properties of the Powders

The storage stability is dependent on the moisture content and water activity of powders, higher values favoring undesirable reactions (microbiological, chemical, enzymatic) that reduce their stability. The a_w_ values as presented in Table 1 are less than the maximum limit value (<0.3), suggesting that the powders were stable. The moisture content of all the tested variants was <5%, which also indicated the high storage stability of the samples.

For producers dealing with powders, the formulation, mixing, packaging, and transportation steps are dependent on the flowability of the powders, which can be characterized by several parameters such as the surface structure, bulk and particle density, size, or shape [26].

Table 1 shows the physical characteristics of the investigated samples. It can be seen that S1 and S3 showed higher BD compared to S2, indicating that preheating of soy proteins prior to encapsulation of the anthocyanins from CCP modified the ability of the molecules to aggregate and consequently the particle size of the capsules, with consequences on powder flow properties [28]. The results are in agreement with those published by Jafari et al. [29], who found that temperature decreased the density of powders with encapsulated anthocyanins, by creating a more porous and fragmented product. The BD of S1 and S3 is almost double compared to S2, higher BD values indicating the protection effect of powders from oxidation [28]. High values for BD also suggest that these types of powders can be stored in small containers [30]. Low BD and TD values for S2 are an indication of larger volume of air trapped in the powder [15]. The HR and CI values collected in Table 1 were used to evaluate the fluency of the microcapsules, results indicating that all the powders have poor flowability.

### 3.2. RE and EE

The TAC of the CCP before encapsulation was 334.26 ± 7.93 mg/100 g d.w. [4]. RE indicates how much of the anthocyanins contained in the CCP are entrapped in the powder after freeze-drying. The RE value calculated for S1, S2 and S3 was 49.34 ± 2.12%, 43.86 ± 3.09% and 46.00 ± 2.87%, respectively. The results obtained in this study are lower compared with previous studies. For example, the encapsulation of anthocyanins from cornelian cherry juice using heated or non-heated soy proteins generated RE values ranging between 51.4 and 76% [19], whereas Robert et al. [31] reported that soy proteins were able to recover over 90% of the anthocyanins from pomegranate juice after the microencapsulation process.

**Figure 1:**
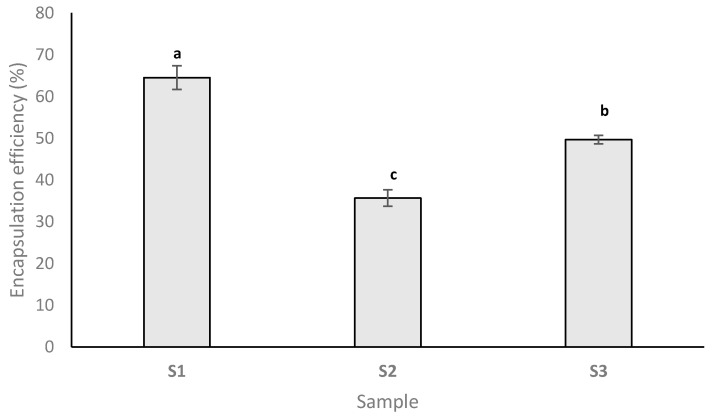
The encapsulation efficiency (EE) of the resulting powders. Columns not sharing the same letter (a, b, c) are significant at *p* < 0.001, based on the post-hoc Tukey test.

EE is a valuable quality parameter that provides information on the potential of the wall material to retain the core material inside the microcapsule [32]. Figure 1 depicts the EE of the resulting powders. It can be seen that when using native SPC for anthocyanins encapsulation, the EE was 64.5 ± 2.83%. In a previous study [19], we focused on encapsulating the anthocyanins from cornelian cherry juice and showed that when using SPC as a coating material and freeze-drying, the EE was 6% higher compared to the result obtained in this study. Lower values were also reported by Robert et al. [31], who encapsulated anthocyanins from pomegranate juice using soy proteins, and only 58.5% of anthocyanins were retained. On the other hand, other studies reported a higher ability of soy proteins to entrap polyphenols. For example, Tumbas Šaponjac et al. [33] obtained an EE of 94.90% for the encapsulation of polyphenols from sour cherry pomace, whereas Robert et al. [31] reported 76.2% for the encapsulation of polyphenols from pomegranate juice. Taking into consideration that usually the extraction of bioactive compounds requires solvents that are not environmentally friendly, the EE obtained in this study shows that similar EE values can be obtained when using CCP without considering a preliminary extraction procedure.

Recently, it was indicated that preheated soy proteins exert a positive effect on the stability of the anthocyanins [34]. Thus, we were interested in testing if preheating soy proteins or the heat treatment of the mixture of soy protein and CCP has a positive impact on the EE of anthocyanins. The effect of two temperatures specific to pasteurization (80 °C) and sterilization (121 °C) usually applied for preservation in the food industry was considered.

As Figure 1 shows, preheating the protein before the encapsulation of anthocyanins did not favor anthocyanins encapsulation, the EE value of S2 representing about 55% of the EE determined for S1. The results are similar to those reported by our research group recently, where preheating the soy proteins at 121 °C had a negative effect on anthocyanins encapsulation [19]. On the other hand, the EE reported for S2 (35.69 ± 1.96%) is about 75% higher compared than the results reported elsewhere [19] for the encapsulation of anthocyanins using preheated soy proteins (20 ± 2%).

Heating the mixture of soy proteins and CCP (S3) generated an EE of 49.68 ± 1.01%. Compared to S2, EE increased by about 39%, and compared to the sample where native soy proteins were used for bioactives encapsulation, EE decreased by about 33%. Taking into consideration the above-mentioned results, we appreciate that when the application allows, the use of CCP rather than juice represents a good alternative for bioactives encapsulation.

Insights into the single molecule-level binding of anthocyanins from cornelian cherries by soybean proteins were gathered by means of the in silico approach. The two major components of soybean proteins are β-conglycinin and glycinin, which are tightly packed globular proteins, exhibiting high stability at thermal treatment [21,22,23]. The β-conglycinin trimer contains three kinds of subunits, namely α’ and α, which share 90.4% homology, and β [21]. The representative models of these proteins were used as receptors in the molecular docking tests for the major anthocyanins of the cornelian cherry fruits.

**Figure 2:**
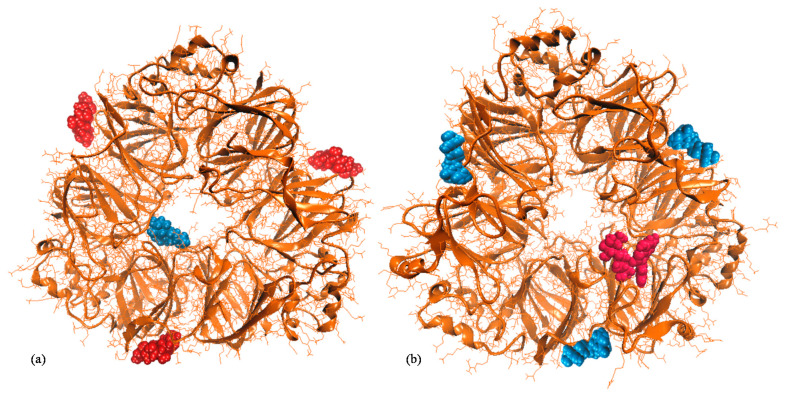
(**a**) Molecular models of the complexes formed between native α’ homotrimer (**a**) and β homotrimer (**b**) of β-conglycinin (New Cartoon style) with cyanidin 3-glucoside (van de Waals style in blue) and cyanidin 3-rutinoside (van de Waals style in red).

Dumitraşcu et al. [23] provided a detailed characterization of the cavities of 7S-α and 11S molecules which are able to accommodate the anthocyanins. The in silico results indicated that the native 7S-α is able to bind three different C3R molecules on small polar cavities with a surface of 480.52 Å² and depth of 7.06 Å, located on the top of each chain of the trimer, outside the protein hollow (Figure 2a). Each C3R molecule is in contact with the Ser^154^, Lys^155^, Lys^162^, Asn^163^, Tyr^165^, Gly^166^, His^167^, Arg^169^, Glu^191^, Asn^193^, Ser^194^, Lys^195^, Asn^257^, Glu^456^ and Gln^457^ residues of a single 7S-α protomer [24]. The most probable C3G binding site (surface of 642.60 Å² and depth of 17.57 Å) is located in the inner space of the 7S-α, involving residues from two different protomers (Arg^185^, Tyr^187^, Asp^208^, Arg^240^, Pro^242^, Ile^265^, Asp^389^, Leu^390^ of chain B, and Asn^183^, Asp^391^, Lys^412^, Ile^483^, Asn^484^ of chain C).

Our in silico tests suggested that the anthocyanins binding by 7S-α is well balanced by 7S-β molecule. Unlike 7S-α, the 7S-β homotrimer is able to accommodate three C3G molecules on the rather deep cavities with a depth of 12.06 Å and surface of 412.34 Å², located on the top of each protomer, outside the trimer hollow (Figure 2b). A close analysis indicated that C3G might establish contacts with Gln^54^, Phe^75^, Val^76^, Leu^77^, Ser^78^, Pro^98^, Gly^99^, Lys^122^, Arg^221^, Ser^254^, Val^276^, Ile^277^, Asn^278^, Glu^279^, Asp^315^, Asn^335^, Phe^336^ and Leu^337^ of each protomer. On the other hand, the C3R binding site is located in the protein hollow and involved amino acids from two adjacent peptide chains (A: Ser^269^, Asn^345^, Asn^346^, Gln^347^ and B: Leu^85^, Val^86^, Asn^87^, Asn^88^, Asp^89^, Asp^90^, Arg^91^, Asp^92^, Tyr^94^, Gln^102^, Arg^103^, Ile^104^, Pro^105^, Ala^106^, Thr^108^, Glu^214^, Asp^215^, Glu^216^, Pro^243^, Gln^244^). Based on the energy values, one can assume that 7S-β exhibits better affinity for both C3G and C3R (binding energy of −34.32 kcal/mol and −23.26 kcal/mol, respectively), compared to 7S-α (binding energy of −27.52 kcal/mol and −13.47 kcal/mol, respectively) [24]. Regarding the 11S fraction from SPI, the contribution to anthocyanins binding is limited to one molecule. As shown previously [23], both C3G and C3R ligands have similar affinity to the same site located in the protein hollow, establishing direct contacts with amino acids from the A, B and C chains of the trimer. The potential protein aggregation events occurring when heating at high temperatures might result in blocking the access to the protein hollow, and further interference with anthocyanins binding to the specific sites. Therefore, the protein aggregation phenomenon, induced by thermal treatment prior to the interaction with the bioactive compounds from CCP, might explain the lower encapsulation efficiency obtained in case of S2 and S3 samples compared to S1.

### 3.3. Color Coordinates

The color coordinates based on L*, a*, b*, chroma and hue angle color measured at different storage periods are collected in Table 2. S3 presented the highest lightness values over the entire storage period considered in this study.

After 30 days of storage, lightness decreased (*p* <0.001) for S1 and S2 and remained constant for S3. The a* values that indicates the tendency toward red were high in all the investigated samples, indicating high anthocyanins content. The biggest differences in terms of a* values measured after 30 days of storage were related to S2. The increased values of a* coordinate was assigned the formation of anthocyanin-derived pigments that stabilized the red-colored flavylium [19]. Immediately after powder production, the lowest b* value was measured for S2 and the highest for S1. Regardless of storage time, the negative values measured for b* coordinate in all samples are associated to slightly alkaline conditions when using soy proteins [33]. Negative b* coordinate values are an indication of blue shades in the sample, whereas positive values suggest a tendency toward yellow. After 30 days of storage, the b* values increased significantly (*p* < 0.001) in all samples. The results are similar to those reported previously, where during storage of powders containing encapsulated anthocyanins, the increase of b* values were associated with the decreased co-pigmentation effects [19,35]. The chroma coordinate of S1 and S3 remained constant during storage and increased significantly (*p* < 0.001) for S2, indicating the highest degree of saturation of the color. Regarding the hue angle, all samples had values closer to 360°, showing that the predominant color was red. During storage, the hue angle registered a slight reduction in the case of all samples.

### 3.4. Microstructure

**Figure 3:**
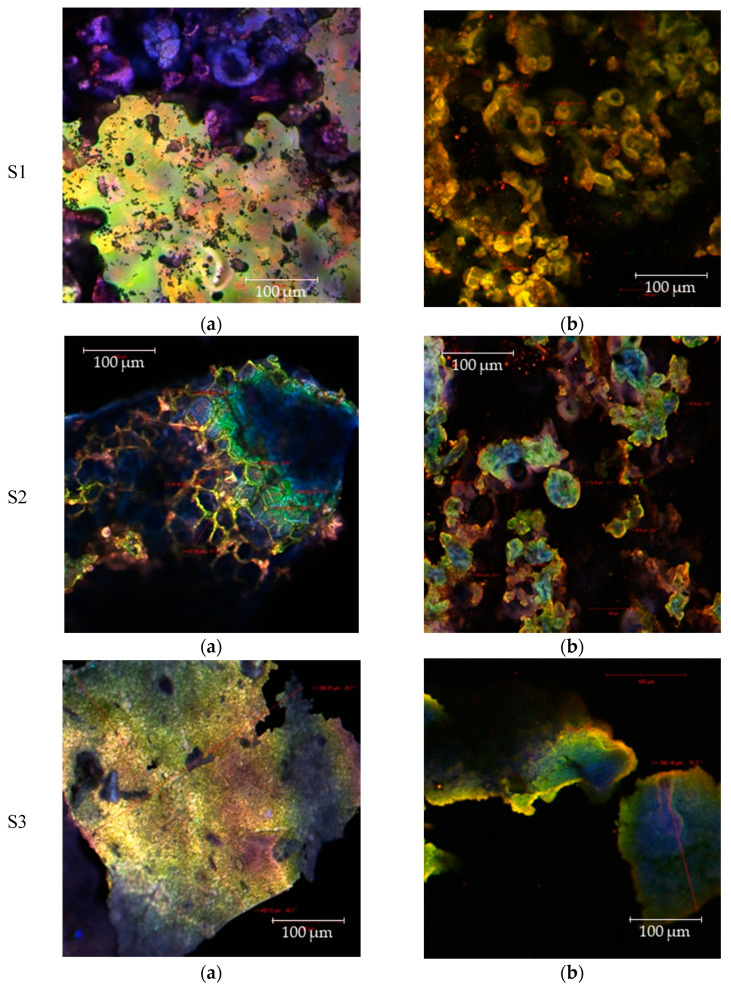
Confocal microscopy images acquired for the native powders (**a**) and for the fluorophore dyed powders (**b**).

Confocal imaging was employed to analyze the microstructure of the resulting powders. In UV light, the maximum absorption of the phenolic compounds was registered at 280 nm, of the carotenoids at 439 to 451 nm and of yellow flavonols or red anthocyanins at ~520 to 580 nm. For the native samples (Figure 3a), the maximum emission was yellow and blue, collected at 600 nm and 480 nm, respectively. For samples labeled with fluorescent Congo Red (Figure 3b), the spectrum was red shifted (550 to 650 nm) due to the complexation of the matrix biopolymers. The encapsulation of the bioactives from CCP using either heated or non-heated soy proteins generated the appearance in the native state of a large scale that delimit large cavities. The microcapsules diameter ranged between 30 and 56 μm in the case of S1, and up to 400 μm for S3. Analysis of the S2 sample by confocal microscopy revealed the presence of an irregular shape matrix with cavities ranging between 20 and 50 μm (Figure 3a). The addition of fluorochromes to S2 generated the most homogeneous appearance of vesicles that varied on average between 30 and 70 μm, and emission that ranged from 480 to 550 nm (Figure 3b). On the other hand, the microcapsules were rigid and did not allow the efficient encapsulation of anthocyanins. Similar results were reported previously, the authors noticing the same pattern when using preheated soy proteins for the encapsulation of bioactives from cornelian cherry juice [19].

### 3.5. In Vitro Digestibility

**Figure 4:**
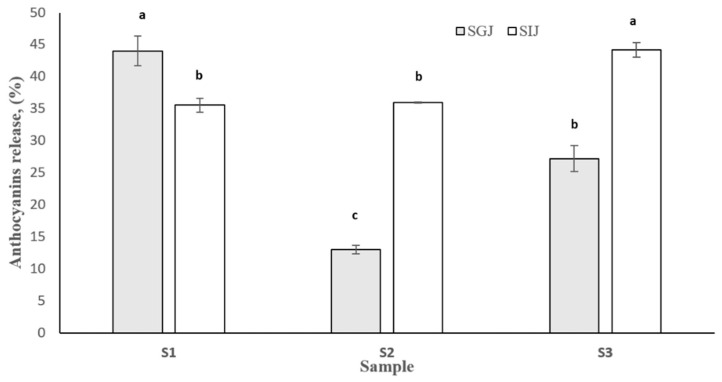
Anthocyanins release after 2 h of incubation with SGJ and SIJ. For the same simulated juice, samples not sharing the same letter (a, b, c) are statistically significant at *p* < 0.05 based on the post-hoc Tukey test.

The stability under simulated GI conditions of encapsulated anthocyanins was investigated by measuring the release of anthocyanins in SGJ and SIJ after 2 h of incubation (Figure 4). The highest anthocyanins release in SGJ (~45%) was obtained when using native SPC (S1) as wall material for anthocyanins encapsulation, and the lowest (~13%) when using preheated soy proteins (S2).

Although the EE was the lowest, it seems that preheating of SPC delivered the protection of anthocyanins needed in SGJ. Compared to S1, heat treatment of SPC solutions allowed a protective effect towards anthocyanins, results that are similar with previous studies [19] where the protective effect was associated with an aggregation event that occurred during heating. In SIJ, the maximum release of anthocyanins was provided by S3, which is ~9% higher compared to S1 and S2. Therefore, it can be concluded that heat treatment provided the stability necessary for anthocyanins to reach the target, results that are similar to those published by Chen et al. [34]. The authors indicated that proper heat treatment of soy proteins is helpful to prevent the loss of anthocyanins.

## 4. Conclusions

This study investigated the potential of encapsulating the anthocyanins from cornelian cherry fruit using heated or non-heated soy proteins. The microcapsule powders had poor flowability. The potential of soy proteins to preserve anthocyanins from cornelian cherry fruit powder was not influenced by heat treatment conditions applied in this study. Using native soy proteins as the wall material delivered the highest encapsulation efficiency and the lowest efficiency was obtained when using preheated soy proteins. The color parameters showed that the predominant color of the powders was red; however, the increase of b* coordinate during storage indicated the decrease of co-pigmentation effects. Regarding the stability of the powders under gastro-intestinal conditions, the microcapsules obtained with non-heated soy proteins were not able to protect the anthocyanins in gastric simulated juice. On the other hand, preheating provided the necessary stability of anthocyanins during gastric digestion as only 13% of anthocyanins from the microcapsules were released. In conclusion, further studies are necessary to identify the optimum heat conditions for soy proteins to allow higher encapsulation efficiency of target compounds.

## Figures and Tables

**Table 1:** The flow properties and water activity (a_w_) of the resulting powders.

Sample	BD (kg/m^3^)	TD (kg/m^3^)	CI (%)	HR	a_w_
S1	213.9 ± 5.9 ^a^	284.7 ± 2.5 ^a^	24.9 ± 1.9 ^b^	1.33 ± 0.03 ^b^	0.17 ± 0.003 ^a^
S2	105.1 ± 5.2 ^b^	166.4 ± 3.5 ^b^	36.8 ± 2.7 ^a^	1.58 ± 0.06 ^a^	0.13 ± 0.001 ^b^
S3	207.5 ± 4.9 ^a^	280.8 ± 3.3 ^a^	26.1 ± 1.7 ^b^	1.35 ± 0.03 ^b^	0.084 ± 0.007 ^c^

S1—powder obtained with native soy proteins and cornelian cherry powder, S2—powder obtained with soy proteins heated at 121 °C and cornelian cherry powder, S3—powder obtained with soy proteins and cornelian cherry powder heated at 80 °C, BD—bulk density, TD—tapped density, CI—Carr index, HR—Hausner ratio. Means on the same column that do not share the same letter (^a^, ^b^, ^c^) are significantly different at *p* < 0.05.

**Table 2:** Color coordinates of the microcapsule powders.

Sample	Storage Time, Days	L*	a*	b*	Chroma	Hue Angle
S1	0	52.08 ± 0.01 ^aC^	21.49 ± 0.06 ^aA^	−4.73 ± 0.02 ^bA^	22.00 ± 0.05 ^aA^	355.52 ± 0.03 ^aC^
	30	51.44 ± 0.02 ^bB^	21.13 ± 0.04 ^bB^	−4.39 ± 0.02 ^aB^	21.58 ± 0.04 ^aB^	355.25 ± 0.01 ^bB^
S2	0	53.17 ± 0.12 ^aB^	21.32 ± 0.11 ^bA^	−6.79 ± 0.01 ^bC^	22.37 ± 0.11 ^aA^	356.96 ± 0.02 ^aA^
	30	46.56 ± 0.49 ^bC^	29.56 ± 0.05 ^aA^	−2.73 ± 0.07 ^aA^	29.68 ± 0.04 ^bA^	349.19 ± 0.3 ^bC^
S3	0	55.21 ± 0.07 ^aA^	19.43 ± 0.06 ^aB^	−5.36 ± 0.02 ^bB^	20.15 ± 0.05 ^aB^	356.46 ± 0.02 ^aB^
	30	55.11 ± 0.01 ^aA^	19.51 ± 0.08 ^aC^	−4.72 ± 0.00 ^aC^	20.07 ± 0.08 ^aC^	355.95 ± 0.02 ^bA^

S1—powder obtained with native soy proteins and cornelian cherry powder, S2—powder obtained with soy proteins heated at 121 °C and cornelian cherry powder, S3—powder obtained with soy proteins and cornelian cherry powder heated at 80 °C. For each color coordinate and sample, values that do not share the same superscript lowercase letter are statistically significant in regard to time at *p*< 0.001. Samples that for each color coordinate and storage time do not share the same superscript uppercase letter are statistically significant at p < 0.001.

## Data Availability

Data sharing not applicable.
